# Implantable Electrochemical Microsensors for In Vivo Monitoring of Animal Physiological Information

**DOI:** 10.1007/s40820-023-01274-4

**Published:** 2023-12-12

**Authors:** Jin Zhou, Shenghan Zhou, Peidi Fan, Xunjia Li, Yibin Ying, Jianfeng Ping, Yuxiang Pan

**Affiliations:** 1 Laboratory of Agricultural Information Intelligent Sensing, College of Biosystems Engineering and Food Science, Zhejiang University, Hangzhou, 310058 People’s Republic of China; 2 ZJU-Hangzhou Global Scientific and Technological Innovation Center, Zhejiang University, Hangzhou, 311200 People’s Republic of China

**Keywords:** Electrochemical microsensors, Implantable sensors, In vivo monitoring, Animal physiological information

## Abstract

In vivo monitoring of animal physiological information plays a crucial role in promptly alerting humans to potential diseases in animals and aiding in the exploration of mechanisms underlying human diseases. Currently, implantable electrochemical microsensors have emerged as a prominent area of research. These microsensors not only fulfill the technical requirements for monitoring animal physiological information but also offer an ideal platform for integration. They have been extensively studied for their ability to monitor animal physiological information in a minimally invasive manner, characterized by their bloodless, painless features, and exceptional performance. The development of implantable electrochemical microsensors for in vivo monitoring of animal physiological information has witnessed significant scientific and technological advancements through dedicated efforts. This review commenced with a comprehensive discussion of the construction of microsensors, including the materials utilized and the methods employed for fabrication. Following this, we proceeded to explore the various implantation technologies employed for electrochemical microsensors. In addition, a comprehensive overview was provided of the various applications of implantable electrochemical microsensors, specifically in the monitoring of diseases and the investigation of disease mechanisms. Lastly, a concise conclusion was conducted on the recent advancements and significant obstacles pertaining to the practical implementation of implantable electrochemical microsensors.

## Highlights

- The materials, fabrication methods, implantation technologies, and applications of implantable electrochemical microsensors for animals were summarized.
- The implantable electrochemical microsensors for monitoring diseases and exploring disease mechanisms were discussed.
- The current status, ongoing challenges, and future development prospects of implantable electrochemical microsensors in monitoring animal physiological information were highlighted.

## Graphical abstract

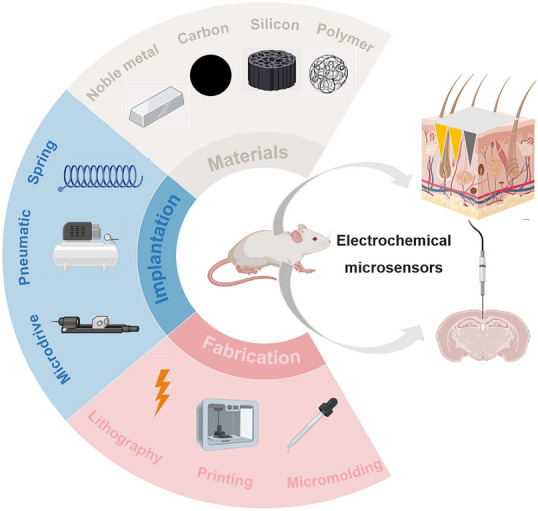

## Introduction

Physiological information serves as a valuable indicator of animals' health status and biological characteristics. Through the continuous monitoring of various indicators, including metabolites, body temperature, and respiratory rate, it becomes possible to detect a wide range of health concerns such as infections, organ impairment, and metabolic disorders. In the field of animal husbandry, the technology for monitoring animal physiology assists farmers and veterinarians in understanding and monitoring the health, behavior, and production performance of livestock. Consequently, it enhances production efficacy, guarantees the exportation of products, and upholds public health and safety [1]. For pet owners, monitoring physiological information about pets can help owners monitor their pets’ health issues more quickly, enhance diagnosis and treatment effectiveness [2]. In the realm of animal research, researchers are capable of grasping information about animal metabolism, and the neurological system, further providing crucial references for researching human diseases. Overall, the advancement of animal physiological monitoring technology will benefit animal health and welfare, encourage the development of animal husbandry and pet health, and provide critical assistance for the research of human diseases.

Animal physiological information encompasses various parameters such as body temperature, heart rate, respiratory rate, blood pressure, weight, exercise ability, and chemicals. The traditional method of monitoring physiological information is the collection of samples for in vitro biochemical, immune, genetic, and other analyses. Techniques such as enzyme-linked immunosorbent assay (ELISA), polymerase chain reaction (PCR), and mass spectrometry are commonly employed in laboratory settings for humoral analysis. These techniques enable accurate early-stage disease diagnosis, leading to reduced medical expenses and improved health outcomes [3]. ELISA and PCR, as gold standards for immunological and molecular diagnostics, are particularly significant in the screening of diseases such as cancer, infectious diseases, genetic and hormonal abnormalities. However, these techniques are still costly and time-consuming, necessitating the use of skilled workers and specialized gear. Researchers are faced with the task of finding alternative diagnostic devices to replace the conventional diagnostic methods. Microsensors have been extensively utilized for monitoring animal physiological information and as auxiliary tools for disease diagnosis, which can be classified as wearable (skin) [4], implantable (tissue) [5], ingestible (gastrointestinal tract) [6] depending on the location of the microsensor application. Wearable sensors detect biomarkers in the biological fluid on the body surface non-invasively, but their accuracy is constrained due to the restricted volume of the biological fluid for detection. Ingestible sensors provide accurate measurement results; however, their long-term presence in the body may pose a threat to animal health. On the other hand, implantable microsensors offer a reliable method for monitoring physiological information that is bloodless, painless, and minimally invasive. Among them, electrochemical sensors have been at the forefront of clinical diagnostics owing to their high performance, portability, simplicity, and low cost [7]. Implantable electrochemical microsensors have been widely employed for monitoring animal physiological information, such as glucose, lactate, dopamine, and biomacromolecules. These sensors assist users in promptly assessing the health status of animals, detecting issues, and implementing suitable actions.

**Fig. 1:**
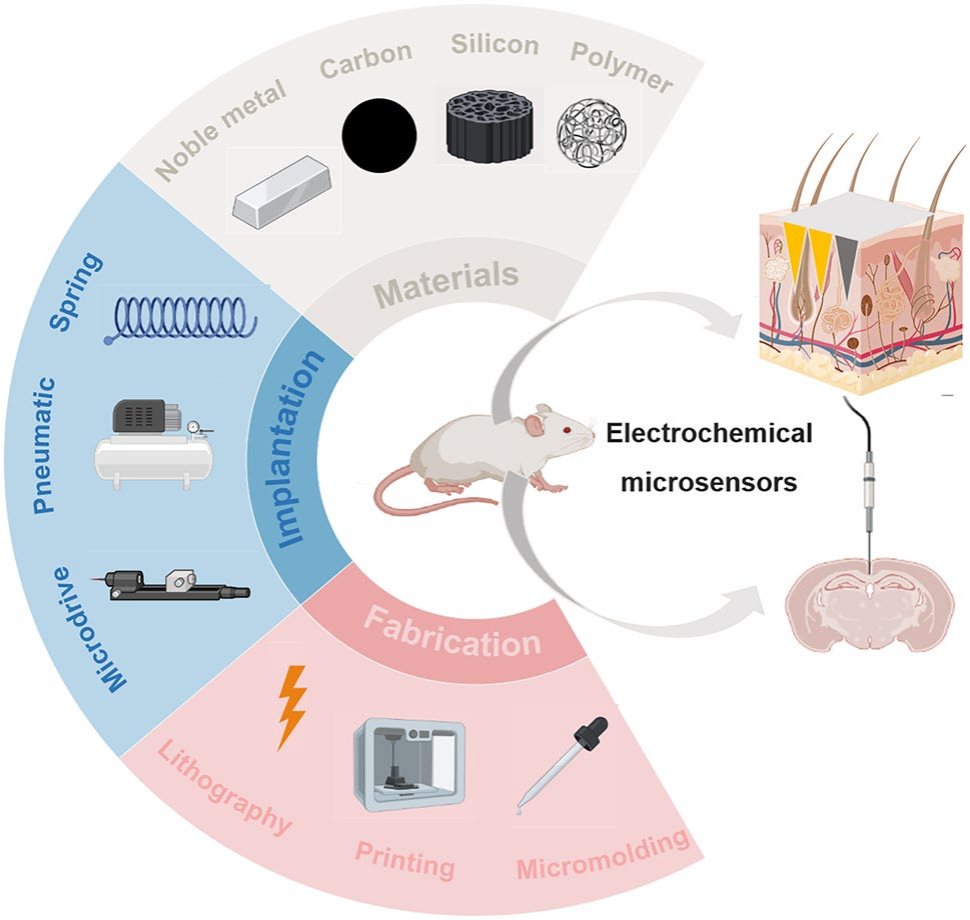
Implantable electrochemical microsensors including materials, fabrication methods, implantation technologies, and reported applications. Created with BioRender.com

This review mainly focuses on monitoring animal physiological information, with a particular emphasis on the widespread use of implantable electrochemical microsensors in this field, owing to their high sensitivity and minimally invasive characteristics. In this review, we comprehensively discussed materials, the fabrication methods, and implantation technologies of microsensors, and innovatively summarized the application of implantable electrochemical microsensors in monitoring physiological information in animals. The development and marketing of implantable electrochemical microsensors require addressing important factors such as long-term durability, compatibility with the human body, reliable power source, and the ability to monitor multi-analytes. The conclusion summarizes the difficulties and potential advancements in the future of implantable electrochemical microsensors for monitoring animal physiological data in vivo (Fig. 1).

## Fabrication of Implantable Electrochemical Microsensors

**Fig. 2:**
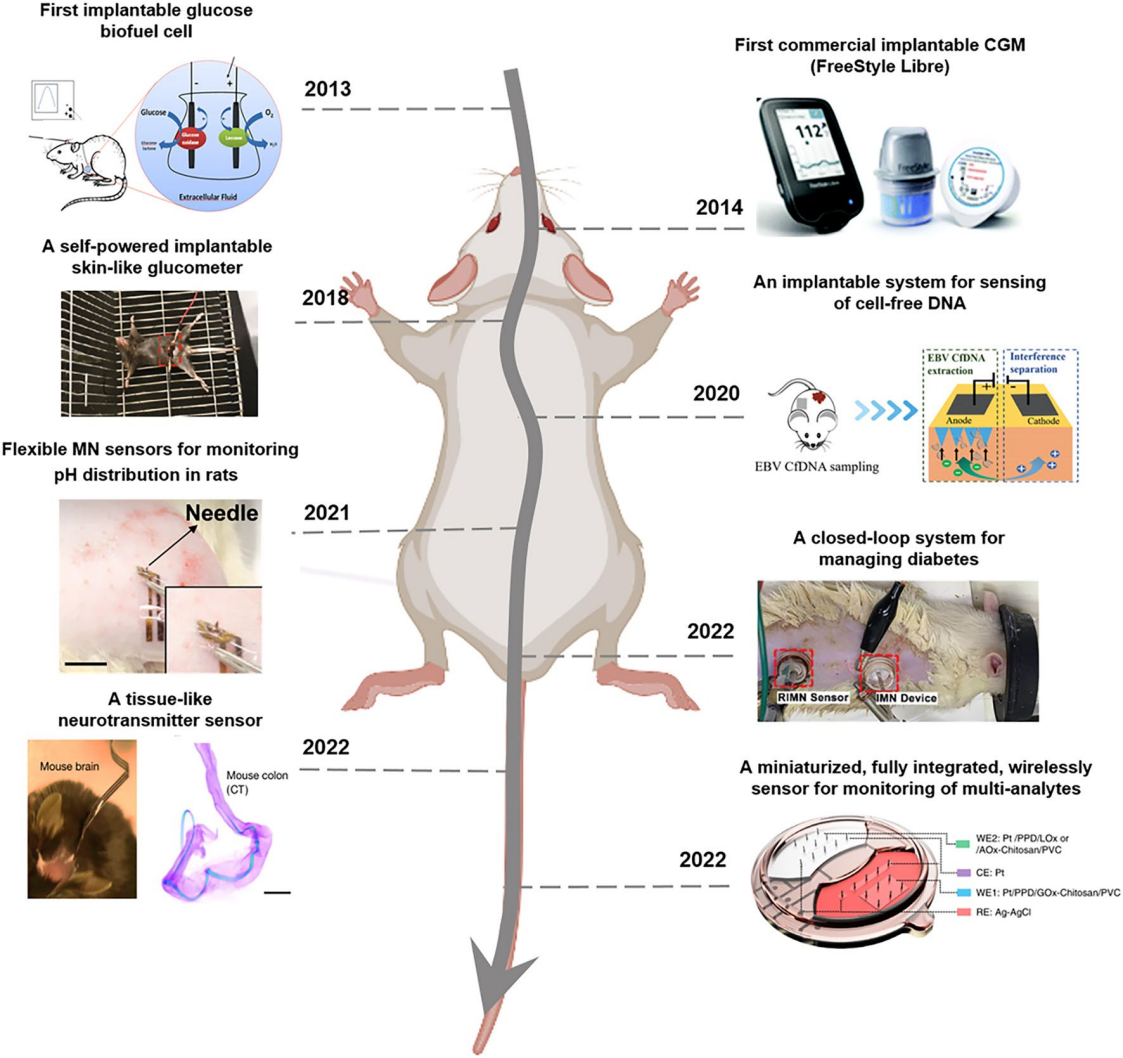
Overview of important developments of implantable electrochemical microsensors in the past decade which include First implantable glucose biofuel cell [9]. Copyright 2013 Springer Nature; First commercial implantable continuous glucose monitor (CGM) [10]. Copyright 2020 Royal of American Society; A self-powered implantable skin-like glucometer [11]. Copyright 2018 Springer; An implantable system for sensing of cell-free DNA [12]. Copyright 2020 Springer Nature; Flexible microneedle (MN) sensors for monitoring pH distribution in rats [13]. Copyright 2021 The American Association for the Advancement of Science; A closed-loop system for managing diabetes [14]. Copyright 2022 John Wiley and Sons; A tissue-like neurotransmitter sensor [15]. Copyright 2022 Springer Nature; A miniaturized, fully integrated, wirelessly sensor for monitoring of multi-analytes [16]. Copyright 2022 Springer Nature

In recent years, significant progress has been made in the field of implantable electrochemical microsensors. Figure 2 illustrates advances in implantable electrochemical microsensors. With the development of materials science, energy and microfabrication, implantable electrochemical microsensors for in vivo monitoring are undergoing a transformation toward miniaturization, multi-analyte sensing, self-powered, and integration of diagnosis and treatment. The choice of materials and fabrication methods plays a crucial role in determining the size, stability, and sensitivity of microsensors [8].

### Materials

The choice of materials is a critical factor that significantly impacts the performance of sensor components. Given the requirement for long-term implantation in *vivo* monitoring, implantable electrochemical microsensors necessitate substrate and electrode materials with enhanced biocompatibility, stability, conductivity, and mechanical strength. These characteristics are essential to ensure the biocompatibility and reliability of long-term monitoring.

#### Substrate Materials

Substrate material serves as the physical supporting structure of the sensor, facilitating the reaction and monitoring processes. In sensor applications, substrate materials are typically required to possess high stability and mechanical strength, while also exhibiting electrical characteristics to ensure optimal sensor performance and accuracy. Rigid substrate materials such as some metals, semiconductors often possess desirable properties such as processability and good mechanical properties [17]. Silicon, for instance, is a type of important substrate material for microsensors due to its excellent semiconductor properties, mechanical strength, and easy processability. Dervisevic et al. designed a high-density silicon-based MN electrochemical sensor for glucose monitoring which shows good performance [18]. Silicon-based MN has good permeability and is easy to penetrate the skin. However, silicon is prone to breakage when implanted under the skin, raising significant biological concerns [19]. Stainless steel substrate is another commonly used metal substrate material, known for its high mechanical strength and machinability, which is easy to be manufactured into a variety of shapes and structures of the sensor. Using stainless steel needles as substrates, Zhang's team fabricated electrochemical microsensors that can be used to wonderfully monitor neurochemicals such as dopamine, norepinephrine and nitric oxide in the rat brain [20–23]. However, the biocompatibility of stainless steel substrate is questionable which can cause skin infections or inflammation.

With the increasing demand for continuous dynamic monitoring in animal physiological information monitoring, flexible materials have gradually attracted attention in various fields due to their advantages such as large surface area, good stretchability, biocompatibility, and wearability [24]. Polymer materials such as polyimide (PI) [25], polyethylene terephthalate (PET) [26, 27], and polydimethylsiloxane (PDMS) [28] are commonly used as substrates for implantable sensors on account of their excellent mechanical properties, thermal stability, and biocompatibility. However, polymer materials have drawbacks such as weaker mechanical strength, limited durability, and complex processing. Another flexible material of interest is hydrogel which exhibits high biocompatibility and has been used in implantable materials [29]. Compared to commonly used material PI, hydrogel processing is simple and efficient. Nevertheless, hydrogel's use in implantable sensors is limited due to its instability and poor mechanical characteristics. Each material has its advantages and disadvantages, so we need to consider the detection object, implantation location, and testing target when choosing a substrate material.

#### Electrode Materials

Electrode materials play a crucial role in the fabrication of implantable microsensors, as their electrochemical performance, biocompatibility, mechanical performance, and processing performance directly affect the conductivity and electrochemical performance of the sensor. Therefore, selecting appropriate electrode material is vital for fabricating high-performance implantable microsensors.

Microsensors made solely of rigid substrate materials have limited sensing performance. In recent years, with advancements in material science and technology, nanomaterials and noble metals have been introduced into the fabrication of microsensors [30]. Noble metals are frequently utilized as electrode materials due to excellent electrical conductivity, high stability, and corrosion resistance [31]. Noble metals, such as gold (Au) and platinum (Pt) are easily micromachined and surface functionalized, making them ideal electrode materials. However, their high cost and potential risks associated with implanting noble metals in animals have hindered their widespread adoption. As alternatives, nanomaterials with tiny sizes, strong conductivity, and stable electrochemical characteristics are often employed as electrode materials in electrochemical microsensors. Meanwhile, nanomaterials have enormous surface areas and controlled chemical structures, providing an abundance of active sites and excellent electrocatalytic effects [32, 33].

Microsensors made of flexible substrate materials often suffer from poor conductivity. To address this issue, researchers have developed stimuli-responsive polymers and conductive polymers based on flexible materials. Stimuli-responsive polymers, known as "smart" materials, can undergo dynamic changes in structure and characteristics (such as conformation, wettability, and surface charge) in response to external stimuli [34]. They can enhance target recognition and modulate electrochemical signals, offering an intelligent electrochemical sensing platform with high selectivity, sensitivity, and controllability for analytical performance. Conductive polymers retain the characteristics of traditional organic polymers, including simpler synthesis, corrosion resistance, and cost reduction. Moreover, one big advantage of conductive polymers compared to many organic polymers is their adjustable chemical structure, which can be modified to change the conductivity of the polymers [35]. The high designability of polymers facilitates the implantation of electrochemical microsensors [36]. Additionally, the surface characteristics and structural features of polymers are compatible with living organisms, making polymer-based microsensors seem to achieve higher histocompatibility and biocompatibility in vivo.

Microsensors need to be implanted to provide long-term monitoring. However, the surface of the sensor may be susceptible to adsorption of certain substances, which can affect its performance. Currently, researchers have developed some anti-fouling coatings to protect the sensor from contamination and improve its long-term stability. In summary, the choice of substrate materials and electrode materials must strike a balance between conductivity and biocompatibility while ensuring the feasibility of long-term stable monitoring.

### Fabrication Methods

Implantable microsensors tend to be applied to different positions and detection targets in animal bodies. In addition to selecting different sensor materials, fabrication methods are also of great significance for meeting different needs. Advanced fabrication methods are required to mass-produce devices with complex geometries and functions [37].

#### Lithography

**Fig. 3:**
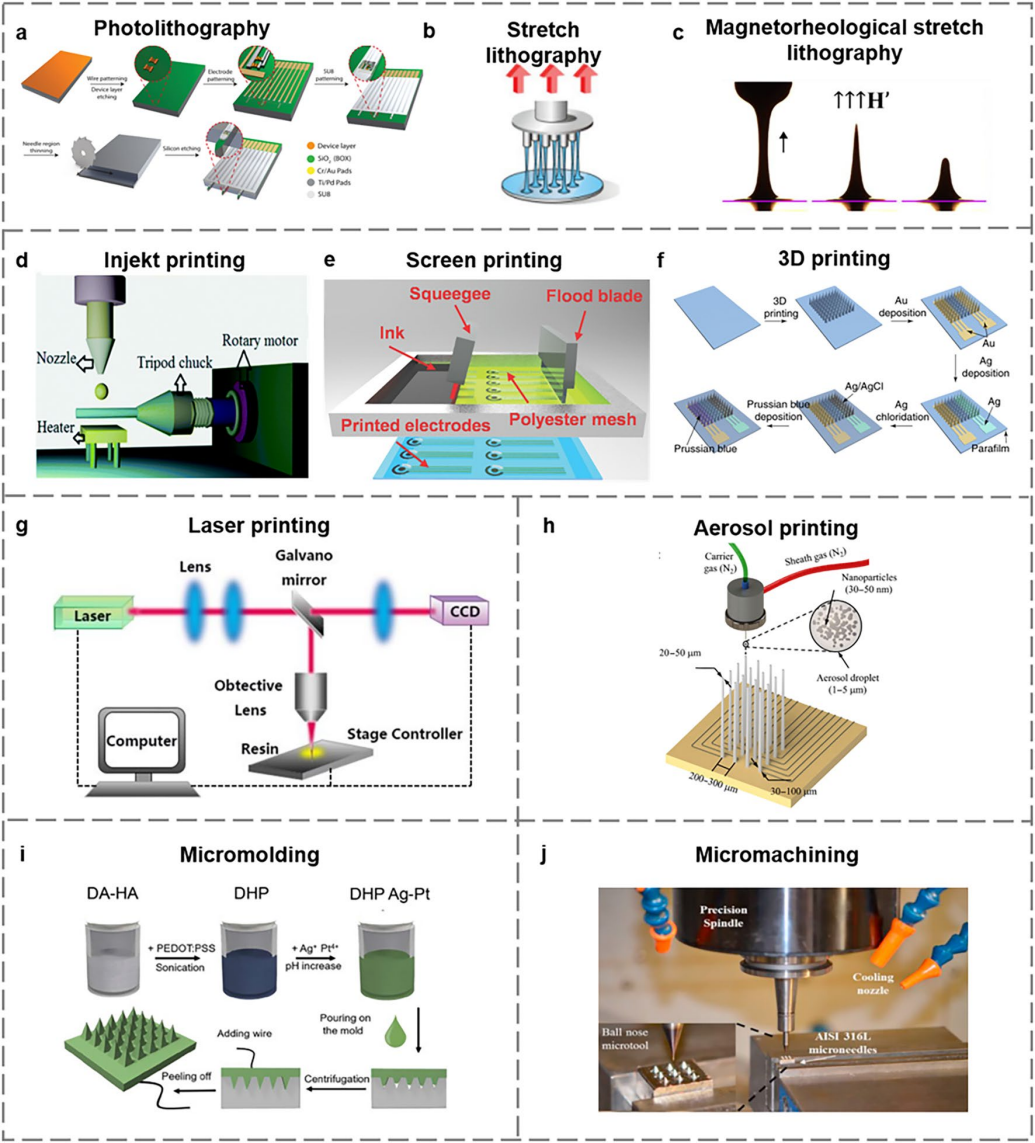
Fabrication methods for implantable electrochemical microsensors. **a** Schematic illustration of electrode fabrication using UV lithography and metal evaporation steps [39]. Copyright 2022 American Chemical Society. **b** Drawing lithography to produce ME [41]. Copyright 2010 John Wiley and Sons. **c** Fabrication of MN using MRDL [42]. Copyright 2018 Elsevier. **d** A schematic of fabricating an implantable microsensor using a rotary inkjet system [46]. Copyright 2018 Royal Society of Chemistry. **e** Screen printing of flexible electrode array on PET substrate [27]. Copyright 2018 John Wiley and Sons. **f** MNA sensor based on 3D printing fabrication [49]. Copyright 2021 Springer Nature. **g** Schematic diagram of a direct laser printing system [50]. Copyright 2019 MDPI. **h** Schematic of aerosol printing of MEA [52]. Copyright 2022 The American Association for the Advancement of Science. **i** HMN-CGM fabrication through micromolding [54]. Copyright 2022 John Wiley and Sons. **j** Schematic illustration of MN fabrication using micromachining [55]. Copyright 2018 Elsevier

One of the commonly used lithography methods is photolithography, which involves exposing photosensitive polymers to ultraviolet (UV) light to create patterns [38]. Harpak et al. manufactured electrodes for protein biomarker detection utilizing UV lithography and metal evaporation processes (Fig. 3a) [39]. Flexible microelectrodes (ME) for measuring functional interactions in multiple brain regions in rats can also be fabricated using conventional photolithography processes and reactive ion etching [40]. However, the limited height of ME produced using conventional photolithography is not conducive to implantation in vivo. To solve this problem, Lee et al. proposed the first stretch lithography method without a mask and light irradiation to produce ME with a high aspect ratio by molding thermosetting polymers (viscoelastic) into specific microstructures through controlled liquid drawing (Fig. 3b) [41]. This method is low-cost but requires high-temperature regulation. Jiang's team proposed a new magnetorheological stretch lithography (MRDL) method of fabricating bionic MN, and microneedle array (MNA). In the proposed method, droplets of curable magnetorheological fluid (CMRF) are extracted directly from almost any substrate to fabricate MN under an external magnetic field. This method not only inherits the advantages of stretch lithography but also eliminates the requirement for temperature regulation (Fig. 3c) [42]. Later, Jiang's team fabricated bionic barbed MN mimicking bee stings based on the MRDL technique, and the barbed structure allowed the MN to easily penetrate the skin [43]. This method is commonly employed for the fabrication of silicon-based sensors. Although lithographic fabrication offers high resolution, it involves complex steps, requires expensive equipment, and incurs high fabrication costs.

#### Printing

Although photolithography methods allow for the fabrication of many devices, the range of materials available for this technique is very limited due to the risk of thermal or chemical degradation of the materials during fabrication. In contrast, printing methods offer more flexibility in terms of material selection and adjustable properties based on the ink used. Common printing methods include inkjet printing, screen printing, 3D printing, laser printing, and aerosol printing [44]. Inkjet printing is an appealing direct writing method that reduces costs and improves efficiency by depositing ink droplets onto various polymer substrates without the need for masks [45]. Pu et al. fabricated an implantable microsensor utilizing a rotary inkjet system on a flexible polyetheretherketone (PEEK) substrate for in vivo glucose monitoring (Fig. 3d) [46]. However, controlling the uniformity of inkjet printing can be challenging, and the range of ink viscosity is limited. Screen printing is a relatively mature method that is achieved by pressing ink through a patterned stencil using a rubber squeegee [47], which is low-cost and suitable for the mass production of ME. The setting of the mesh on the screen will affect the shape and size of the printed electrode. To achieve high-precision printing, it is necessary to consider the shape and size of the mesh. Jin et al. prepared metal-organic framework (MOF)-modified sensors based on screen printing to achieve continuous in vivo monitoring of ascorbic acid, glycine, l-tryptophan (l-Trp), and glucose (Fig. 3e) [27]. Both inkjet printing and screen printing can only fabricate microsensors on a flat surface, three-dimensional (3D) printing offers the possibility to develop sensors on non-planar substrates [48]. Liu et al. fabricated an integrated MN biosensing device through 3D printing for in vivo monitoring of glucose in mice (Fig. 3f) [49]. Although 3D printing is a versatile fabrication method in terms of geometry, spacing, height, and number of ME, it prints microsensors with limited resolution, which may not be conducive to in vivo sensing. Laser printing is a cost-effective method for fabricating conformal electronics without the need for vacuum or masks (Fig. 3g) [50]. Unlike conventional printing processes such as inkjet, laser printing allow printing with highly viscous inks, but the size of the deposited features is restricted by the size of the laser beam. Aerosol printing is a non-contact printing method that involves spraying processed nanoscale metal and ceramic particles in aerosol form onto the substrate surface to create microscale structures [51]. It offers high accuracy (~ 10 μm), controllable printing height, and compatibility with various materials. Mohammad et al. designed a microelectrode array (MEA) for electrophysiological recording in the mouse brain based on aerosol printing (Fig. 3h) [52]. However, this method requires specialized equipment and incurs high processing costs.

#### Others

Micromolding is a simple and low-cost microfabrication method that involves pouring molten or liquid materials into a micro-mold to create the final product [53]. In a study by GhavamiNejad et al., a hydrogel microneedle (HMN)-CGM was successfully fabricated using micromolding (Fig. 3i) [54]. Compared to other methods, micromolding has a lower resolution. Another fabrication method is micromachining which generates the final structure on a machine tool by removing raw materials to the desired geometric shape. Metals are the most commonly utilized materials for micromachining (such as stainless steel (Fig. 3j) [55]). In general, the choice of materials and fabrication procedures for implantable electrochemical microsensors may vary depending on the specific application, but it is crucial to ensure excellent sensing capabilities and biocompatibility.

## Implantation Technologies for Electrochemical Microsensor

Implantable electrochemical microsensors are designed to be minimally invasive and remain in animals for extended periods, enabling prolonged monitoring and disease management. However, the sharp edges of microsensors can harm surrounding tissue and create long-term stress on the adjacent biological environment [56]. Furthermore, the constituent materials of the microsensor [57] and the implantation speed [58] also affect the stresses that the microsensor exerts on the tissue. Traditional implantation techniques, such as manual implantation or syringe-based implantation are simple and fast [59, 60]. However, the force applied during manual implantation is challenging to control, resulting in inefficient microsensor implantation and potential tissue injury around the implantation site. To address these challenges, researchers have developed innovative implantation technologies that enable long-term microsensor implantation with minimal damage.

### Spring-Loaded Implantation

**Fig. 4:**
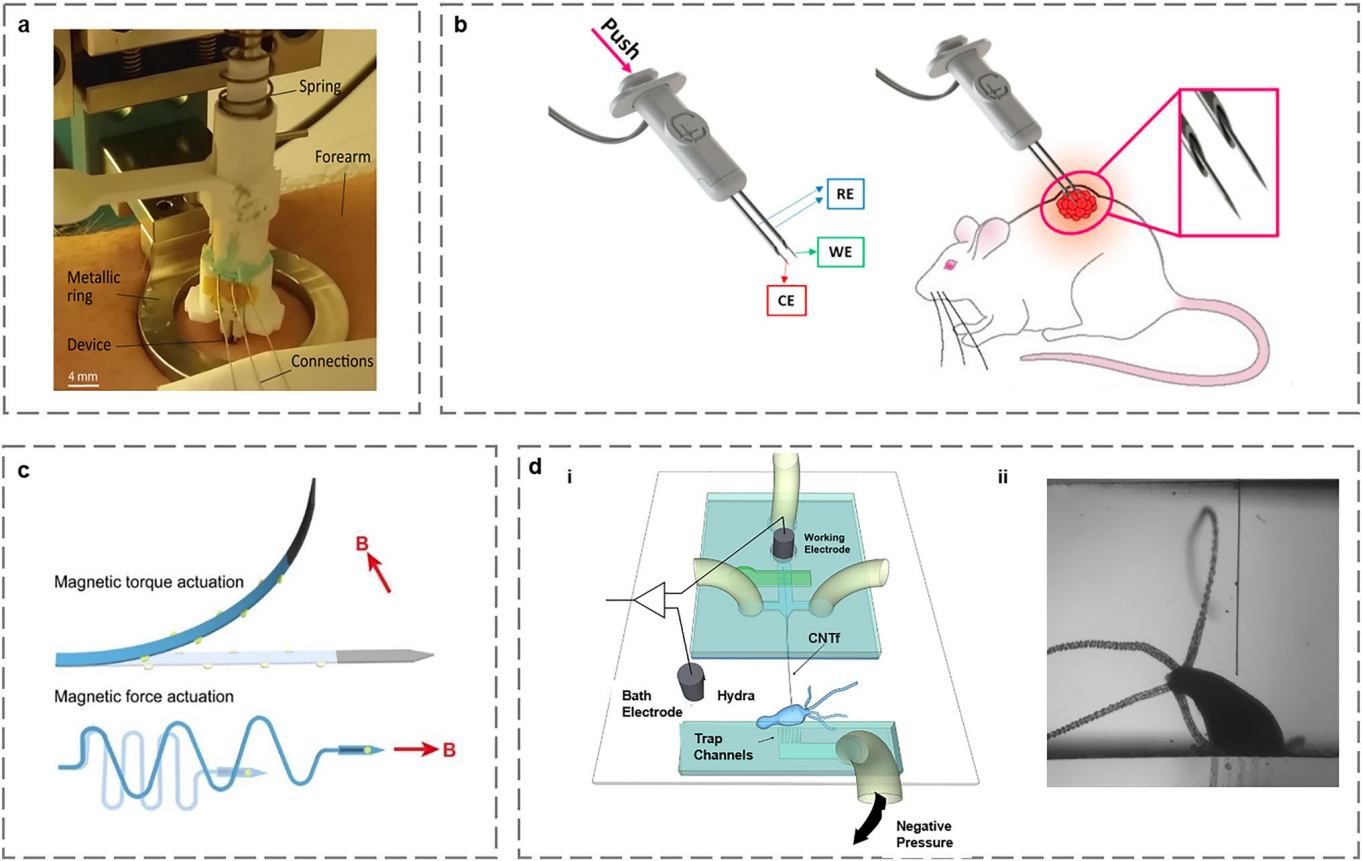
Schematic diagram of different implantation technologies of implantable electrochemical microsensors. **a** Schematic of a spring-loaded mechanism [61]. Copyright 2018 Springer Nature. **b** Schematic illustration of electrodes pushed into a tumor by a spring-loaded device [63]. Copyright 2020 Elsevier. **c** A schematic representation of the magnetic implantation device [67]. Copyright 2019 American Chemical Society. **d** Microfluidic drive implantation of CNT fiber in the *Hydra.* (i) Schematic of Hydra recording electrophysiology chamber. (ii) ME located next to *Hydra* [70]. Copyright 2018 American Chemical Society

Spring-loaded implantation utilizes mechanical springs, which deform under external forces and return to their original state when the pressure is removed. To achieve proper skin penetration, Ribet et al. designed a custom-implantable device that included a spring-loaded mechanism (Fig. 4a) [61]. When the spring is fully contracted, the implanted device exerts an initial force of 5 N, much greater than the force required to penetrate the skin with minor damage [62]. The above equipment is not integrated. Miripour et al. developed a biopsy-guided probe sensor for H_2_O_2_ detection in tumors, with probes and spring integrated into a 3D printed sensor housing (Fig. 4b) [63]. The spring-loaded implantation technology is easy to implement and is already commercially available.

### Pneumatic Implantation

Earlier in 1992, Patrick et al. constructed a pneumatic implantation system consisting of an implantation device and a support device connected to the support device via a plastic tube [64]. The MEA was initially placed on the end piece by employing surface tension from a drop of saline. The support device then emits an air pulse, accelerating the piston toward the end piece. Finally, the momentum of the piston is transferred to the end piece which is then implanted into the tissue. The implantation velocity can be varied by changing the air pressure and piston mass to achieve a controlled high insertion velocity. Chauvière et al. compared results obtained with surgical implantation and vacuum implantation of floating silicon neuro probes into the cortex of non-human primates [65]. The results showed that vacuum implantation using the pneumatic method yielded better-quality neural signals. The pneumatic implantation technology allows for controlled velocity implantation and reduced tissue damage but requires additional equipment to provide air pressure, which is inconvenient to use.

### Microdrive Implantation

#### Magnetic Drive Implantation

Both spring-loaded and pneumatic implantation technologies generate a certain compressive force on the electrode to implant it into animals, which tends to bend or even break when the electrode is flexible or very small in size. To address this issue and preserve the integrity of the electrode, researchers have explored the use of a magnetic field to implant electrodes with ferrous metal tips into animals [66]. Gao et al. developed a remotely driven implantation method using a magnetic field, where a flexible ME with electrodeposited iron and nickel at the tip formed a magnetic head. The implantation angle and speed were adjusted using a permanent magnet (Fig. 4c). Immunohistochemical staining results showed that the implantation caused little damage in the mouse brain, opening up new avenues for controlled and minimally invasive brain connectivity in vivo [67].

#### Electric Drive Implantation

Another approach is electric drive implantation, which utilizes motors to electronically drive microdrives. Fee et al. demonstrated a motorized, small microdrive for the implantation of electrodes in a bird’s brain to record the physiological information of the brain [68]. The whole process was remotely controlled, eliminating the need to disturb the animal. This microdrive was successfully used in the spotted-breasted meadowlark without capturing or restraining the bird. The motor offers greater precision and controllability of movement than manual implantation. With the development of microscale electro-mechanical system (MEMS), Pang et al. designed a new electrolytic parylene balloon microdrive using MEMS technology [69]. The microdrive consists of an electrolytic electrode located on a silicon spring structure, with electrolytes filled in the parylene balloon. Electrolysis generates gas, building pressure inside the balloon, which expands and stretches the spring, thereby pushing the microsensor forward. Electric drive often requires very little power and is less likely to create resistance, but it does generate heat.

#### Microfluidic Drive Implantation

Microfluidic drive implantation is another technology that addresses the challenges posed by flexible sensors. Many microsensors are made of flexible materials and are susceptible to bending during implantation. Based on this, Vitale et al. developed a microfluidic microdrive that utilizes viscous fluid flow to maintain tension in the electrode structure [70], thus preventing an increase in the thickness or stiffness of the electrodes during implantation (Fig. 4d–i). With the device, carbon nanotube (CNT) fiber was successfully implanted in the cnidarian *Hydra (H. littoralis)* (Fig. 4d–ii). In addition, microfluidic drives can precisely drive the electrode position with micron-level precision.

Traditional manual or surgical implantation methods can cause significant tissue damage and imprecise placement of microsensors. To address these issues, spring, pneumatic, and microdrive-based implantation technologies have been developed. However, pneumatic and microdrive implantation technologies that utilize electric and magnetic forces require additional equipment support, are challenging to operate, and generate heat that may harm surrounding tissue. Spring-based implantation technology is commercially available, integrated, and user-friendly, which can be used for the implantation of rigid microsensors. In terms of flexible microsensors, microfluidic-driven implantation technology has a promising future. In a word, researchers need to choose the appropriate implantation technology based on the application scenario and sensor materials.

## Application of Implantable Electrochemical Microsensors for In Vivo Monitoring of Animal Physiological Information

Implantable electrochemical microsensors have the capability to monitor physiological information in animals in real time, including metabolites, biomacromolecules, electrolytes, neurotransmitters, and oxidative stress products. These sensors hold significant potential for monitoring diseases (Table 1) and exploring disease mechanisms (Table 2). In the field of monitoring diseases, these sensors enable real-time monitoring of biochemical parameters within organisms, such as pH value and ion concentration thereby promptly detecting early signs of diseases and providing more accurate diagnosis and treatment guidance [71]. In terms of exploring disease mechanisms, these sensors can provide more detailed internal information about organisms, aiding researchers in gaining a deeper understanding of the mechanisms underlying diseases. By monitoring changes in specific biomarkers, the pathological processes and molecular mechanisms of diseases can be revealed. As a result, these sensors can be utilized in the study of various diseases, such as cancer and neurological disorders.

**Table 1:** Summary of progress on implantable electrochemical microsensors for monitoring diseases in animals

Analyte	Electrode		Modification materials		Method		Linear range		LOD		Stability		Refs.	
Glucose	Solid MN		Au/pTCA/GOx/Nafion		CA		0.05–20 mM		19.4 μM		3 days		[83]	
	Solid MN		Au/PB/GOx/Nafion		CA		0.8–24 mM		8.65 μM		7 days		[49]	
	Solid MN		Au /Fc-PAMAM/GOx		CA		1–9 mM		660 μM		/		[18]	
	Pt ME		Pt/GOx-BSA-GA/Nafion		CA		up to 14 mM		/		4 days		[61]	
	Au ME		Au/GOD/BSA		CA		3–13 mM		920 μM		7 days		[84]	
	Solid MN		Au/Pt black/Nafion		CA		1–20 mM		22.5 μM		7 days		[87]	
	HMN		DA-HA/PEDOT: PSS/Ag-PtNPs		CA		/		900 μM		14 days		[54]	
Lactate	Solid MN		Au/Au-MWCNTS/pMB/FADGDH		CA		0.01–0.2 mM		2.4 μM		30 days		[90]	
	Pt MN		Au-NPs/PDA-NSs		DPV		0.375–12 mM		50 μM		/		[92]	
Glucose	MEA		GOx/PtNPs/PANI		CA		2–12 mM		260 μM		7 days		[94]	
Uric acid			UOx/PtNPs/PANI				0.1–1.2 mM		4 μM					
Cholesterol			ChOx/PtNPs/PANI				1–12 mM		440 μM					
Glucose	MN		Pt/PPD/GOx-Chitosan/PVC		CA		0–40 mM		320 μM		0.5 day		[16]	
Lactate			Pt/PPD/LOx-Chitosan/PVC				0–28 mM		120 μM					
Alcohol			Pt/PPD/AOx-Chitosan/PVC				0–100 mM		500 μM					
Protein	CP		E200acryl-CP/catechol-agar		CA		0.1–0.5 mg/mL		/		/		[97]	
	Solid MN		Au /anti-HER2		DPV		0.00001–0.00025 mg mL^−1^		0.048 μg mL^−1^		/		[98]	
cfDNA	AuMN		Graphene/PBA/BSA/dRNP		CA		/		1.1 fM		10 days		[28]	
pH	Solid MN		Au/PANI/ PMC3A		OCP		/		/		/		[13]	
	HMN		DA-HA/PEDOT: PSS		CA		3.5–9		/		5 days		[103]	
Cl^−^	CF		OxGO/Ti_3_C_2_T_x_/Ag		DPV		/		10 μM		/		[104]	
Ca^2+^	CF		Ca-ISM		OCP		/		1 μM		/		[106]	
Na^+^	SPE		PEDOT: PSS/ISM		OCP		0.75–200 mM 1–128 mM 0.25–4.25 mM 5.5–8.5		/		30 days		[107]	
K^+^														
Ca^2+^														
pH														
pH	ISE		SST/C/f-MWCNTs/ISM		CP		5.0–8.5		/		/		[60]	
K^+^							0.032–10 mM		15 μM					
Na^+^							0.032–32 mM		18 μM					
Ca^2+^							0.032–32 mM		3.9 μM					
Li^+^							0.032–10 mM		12 μM					
Cl^−^							0.01–32 mM		8.4 μM					
H_2_O_2_	CF		HCT@GNSs-PtNPs		CA		up to 6 mM		0.05 μM		42 days		[108]

**Table 2:** Summary of progress on implantable electrochemical microsensors for exploring disease mechanisms and others

Analyte	Electrode	Modification materials	Method	Linear range	LOD	Stability	Refs.
DA	CF	/	CA	/	/	/	[117]
	CF	PTA-PANI	CA	5–30 µM	/	/	[119]
	Pt wire	AuNPs/rGO	DPV	/	16.57 nM	7 days	[122]
	Stainless steel needle		CA	0.001–10 μM	0.37 nM	7 days	[20]
Serotonin	CF	Nafion	FSCV	/	/	/	[124]
	Graphene	Graphene/iron oxide NP@SEBS	CA	/	3.5 nM	112 days	[15]
Glutamate	NdNiO_3_ (NNO) thin film	NdNiO_3_ (NNO)/Nafion/GluOx	CA	1–700 μM	16 nM	/	[125]
L-glutamate GABA	Pt	Pt black/GluOx(GABase)/mPD	CA	/	120 nM	20 days	[126]
					40 nM		
Choline	Ag/AgTPB	1,2-DCE containing 5 mM THATPB	CA	1–54 µM	370 nM	/	[127]
H_2_O_2_	CF	EOGO/Au/o-Cl-DBP	DPV	0.5–600 μM	36 nM	10 days	[128]
	CF	PtNP-NPG	CA	0.2–200 µM	10 nM	/	[141]
NO	CF	CNF/hemin/Nafion	DPV	0.025–1 µM	10 nM	/	[129]
	CF	Nickel porphyrin/fluorinated xeroge	CA	/	12.1 ± 3.4 nM	/	[130]
O_2_^•−^	CF	CNT/PBNPs/SIL/SOD	CA	1.0–228 μM	1200 nM	7 days	[132]
ONOO^−^	CF	HEMF	DPV	0.02–2 µM	12.1 ± 0.8 nM	6 days	[134]
O_2_	CF	Co/N/C	CA	/	/	/	[136]
	CF	Pt nanocatalysts@SNM/OEG	CA	/	/	14 days	[137]
L-Dopa	CP	CP/TYR/Nafion	SWV	0.5–3 μM	500 μM	/	[138]
			CA	0.25–3 μM	250 μM		
Fentanyl	Pt ME	Graphene ink/4 (3-butyl-1-imidazolio)-1-butanesulfonate) ionic liquid	SWV	20–160 μM	27800 µM	/	[139]

### Metabolites

Metabolites are metabolic intermediates that are catalyzed by several enzymes found inside the cell [72]. By measuring and analyzing metabolites, we can gain a better understanding of the metabolic status of animal bodies, which helps in identifying health problems early and taking corresponding measures for prevention and treatment. In this section, we will discuss some important developments in implantable electrochemical microsensors for monitoring different metabolites.

#### Glucose

Glucose is the main source of energy for cellular activity in living organisms. Glucose levels in pigs are significantly affected by age, and young mini pigs are prone to hypoglycemia [73]. Similar to humans, when pigs suffer from sustained hypoglycemia, brain cells are irreversibly damaged, leading to brain death and even sudden death [74]. Long-term hyperglycemia can cause various complications, including cardiovascular complications (stroke) [75], neuropathy [76], and diabetic foot ulcers [77]. Therefore, long-term management and control of diabetes are essential. Glucose concentrations in interstitial fluid (ISF) correlate with glucose concentrations higher than those in other biological fluids [78]. CGMs have been introduced in recent years and are being adopted by an increasing number of patients. These systems use a glucose sensor whose tip is inserted into the subcutaneous adipose tissue under the skin to measure glucose in ISF in real time [79]. Commercially available CGMs, such as the Abbott FreeStyle Pro with a length of 5 mm implantable probe [80], can cause discomfort and even adverse reactions to the user with long-term implantation. In comparison to conventional implantable probes, MN (typically shorter than 1 mm in length) can be implanted under the skin painlessly and bloodlessly. In addition, MN-based CGM sensors have less biological contamination, lowering the risk of skin infection. Furthermore, MN has a higher electrode surface area and demonstrates great sensitivity in both in vitro and in vivo tests [81].

**Fig. 5:**
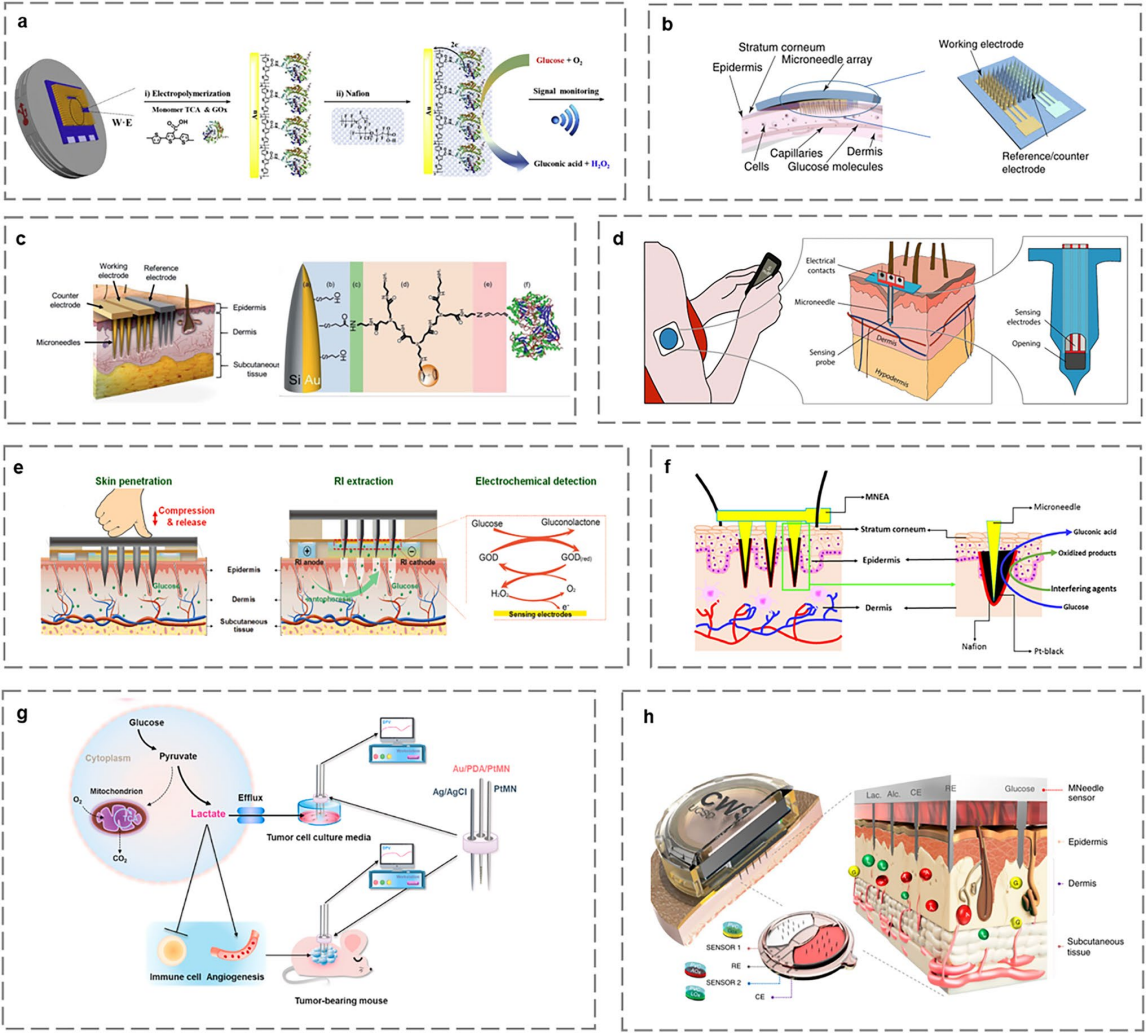
Schematic diagram of MN-based electrochemical microsensors for monitoring metabolites. **a** Fabrication of gold-coated MN-based microsensor [83]. Copyright 2018 Elsevier. **b** MNA based on 3D printing [49]. Copyright 2021 Springer Nature. **c** Implantation of MN-based microsensor into the skin and modification of the working electrode [18]. Copyright 2021 John Wiley and Sons. **d** Silicon hollow MN integrated with sensing electrodes [61]. Copyright 2018 Springer Nature. **e** Transdermal monitoring strategy of glucose sensors [84]. Copyright 2022 Elsevier. **f** Implantation of the MEA into the rat skin and direct oxidation of the glucose on Pt black surface [87]. Copyright 2021 Elsevier. **g** Schematic illustration of Pt ME in vivo monitoring of lactate [92]. Copyright 2021 Elsevier. **h** A wearable sensor based on multiplexed MNA [16]. Copyright 2022 Springer Nature

MN-based electrochemical microsensors for glucose monitoring can be categorized into two main types: enzymatic sensors and non-enzymatic sensors [82]. Enzymatic glucose electrochemical microsensors rely on monitoring oxygen consumption based on enzyme-catalyzed reactions. Kim et al. electropolymerized terthiophene carboxylic acid (TCA) and immobilized glucose oxidase (GOx) on Au-coated MN to form sensing electrodes (Fig. 5a) [83]. Liu et al. developed a CGM device using a 3D printing microfabrication step (Fig. 5b) [49]. It continuously monitored subcutaneous glucose levels in mice (normal and diabetic), and obtained highly correlated results with commercial CGMs. Dervisevic et al. presented the first electrochemical microsensor based on high-density silicon MN coated with Au and then modified with conjugated dendrimer containing a redox mediator (ferrocene-nucleated poly(amidoamine) dendrimer [Fc-PAMAM]) and a catalytic bioreceptor GOx (Fig. 5c) [18]. Successful application of the biosensor in mice demonstrated that MN-based microsensors are capable of monitoring changes in ISF glucose levels. In the aforementioned examples, sensitive materials are utilized to modify MN so that they can be directly used as sensing electrodes. Another technique involves integrating MN with sensing electrodes. Ribet et al. used standard silicon microfabrication methods to integrate ultra-miniaturized electrochemical sensing electrodes in a single hollow MN lumen (Fig. 5d). This miniaturization made the system significantly less invasive than commercial CGMs, while still maintaining satisfactory sensitivity and physiologically expected values of measurement latency [61]. Chen et al. investigated an electrochemical microsensor for monitoring glucose in ISF (Fig. 5e) [84]. Solid MNA was used to pierce the skin and form an ISF channel. ISF was extracted from the skin's surface using a mild current through a reverse iontophoresis (RI) unit, which boosted the extraction volume by around 1.6 times. Final data were gathered by wireless transmission to a cell phone via a sensing device. The innovative sampling method of the microsensor provides research ideas for monitoring other biomarkers in ISF.

Enzymatic sensors are widely used for long-term monitoring of glucose. However, factors such as temperature, pH, and humidity can affect enzyme stability, leading to reduced monitoring accuracy [85]. On the other hand, non-enzymatic sensors offer various advantages, including stability, ease of manufacturing, reproducibility, and freedom from oxygen constraint [86]. Non-enzymatic sensors are based on the direct electrochemical oxidation of glucose, which is tracked through an electrocatalytic dehydrogenation process. For instance, Chinnadayyala et al. developed a non-enzymatic microneedle electrode array (MNEA) based on highly porous Pt black modified with a Nafion-filled MN surface to reduce biostasis (Fig. 5f). This MNEA remained stable in rats for approximately 7 days and is expected to be used in wearable devices [87]. GhavamiNejad et al. innovatively developed an HMN-based non-enzymatic electrochemical microsensor using dopamine (DA)-hyaluronic acid (HA) hydrogel combined with poly (3,4-ethylenedioxythiophene) polystyrene sulfonate (PEDOT: PSS) and Ag-Pt nanoparticles (Ag-PtNPs) for real-time and non-enzymatic monitoring of glucose in ISF [54]. PtNPs and AgNPs were utilized as electrocatalysts to oxidize glucose after the in situ reduction in Pt ions in the presence of the DA's catechol fraction. The sensor successfully tracked glucose levels in diabetic rats and has promising potential for long-term glucose monitoring.

#### Lactate

In animals, lactate is a metabolic compound produced by proliferating cells and during anaerobic conditions such as strenuous exercise [88]. Excess lactate leads to inadequate tissue perfusion and increased anaerobic metabolism, which may result in hyperlactatemia and severe metabolic dysregulation [89]. Therefore, monitoring lactate levels is crucial for diagnosing and assessing health problems associated with lactic acidosis. Bolella et al. developed an electrochemical microsensor based on AuMN, using nanocarbon and lactate oxidase (LOx) functionalizing AuMN [90]. The sensor was experimented in human serum and demonstrated its utility in sports medicine and clinical monitoring. In addition to manufacturing lactate through exercise, cancer cell metabolism, increases glucose consumption through glycolysis, resulting in the production and continuous export of lactate from the cells. Consequently, elevated lactate levels in the extracellular microenvironment are considered a sign of cancer [91]. Li et al. reported a non-enzymatic electrochemical microsensor based on PtMN electrodes (Fig. 5g) that enabled in vivo recording of the characteristic voltammetry signal associated with lactate in living tumors, which was positively correlated with tumor load and growth [92].

#### Multi-Metabolites

Although a single metabolite can be used to diagnose a health disorder, there are still issues that cannot be accurately predicted. Simultaneous measurement of several metabolites can reduce the false negatives in clinical diagnosis that are more likely to occur when measuring individual metabolites [93]. Gao et al. showed a flexible microneedle electrode array-based biosensor (MEAB) for simultaneous monitoring of glucose, uric acid, and cholesterol with high sensitivity and wide linear range, possessing the potential for the development of blood metabolite monitoring at home [94]. Tehrani et al. designed a sensor based on an integrated wearable MNA for painless, continuous, real-time monitoring of glucose and lactate or glucose and alcohol [16]. The sensor collected molecular-level electrochemical signals from the wearer's ISF through the insertion of microneedle tips into the epidermis (Fig. 5h).

It is vital to use implantable MN-based sensors to monitor metabolites in animals. Regular monitoring of metabolite levels can help pet owners and livestock farmers detect early signs of potential disease in animals and take preventive measures to reduce the risk of disease. Additionally, monitoring model animals is highly beneficial for exploring certain disease mechanisms.

### Biomacromolecules

Biomacromolecules, such as proteins, nucleic acids, and polysaccharides, are large molecules found in the cells of living organisms. These biomacromolecules are essential components that make up animal cells, tissues, and organs. The levels of biomacromolecule synthesis and metabolism have a direct impact on human health. Biomacromolecules are also closely related to the occurrence and progression of animal diseases. In certain diseases, specific proteins or metabolites can exhibit abnormalities. For example, in many cancers, the tumor itself and the changing cells release abnormal proteins or metabolites, which can be used to diagnose and monitor disease progression [95].

#### Proteins

**Fig. 6:**
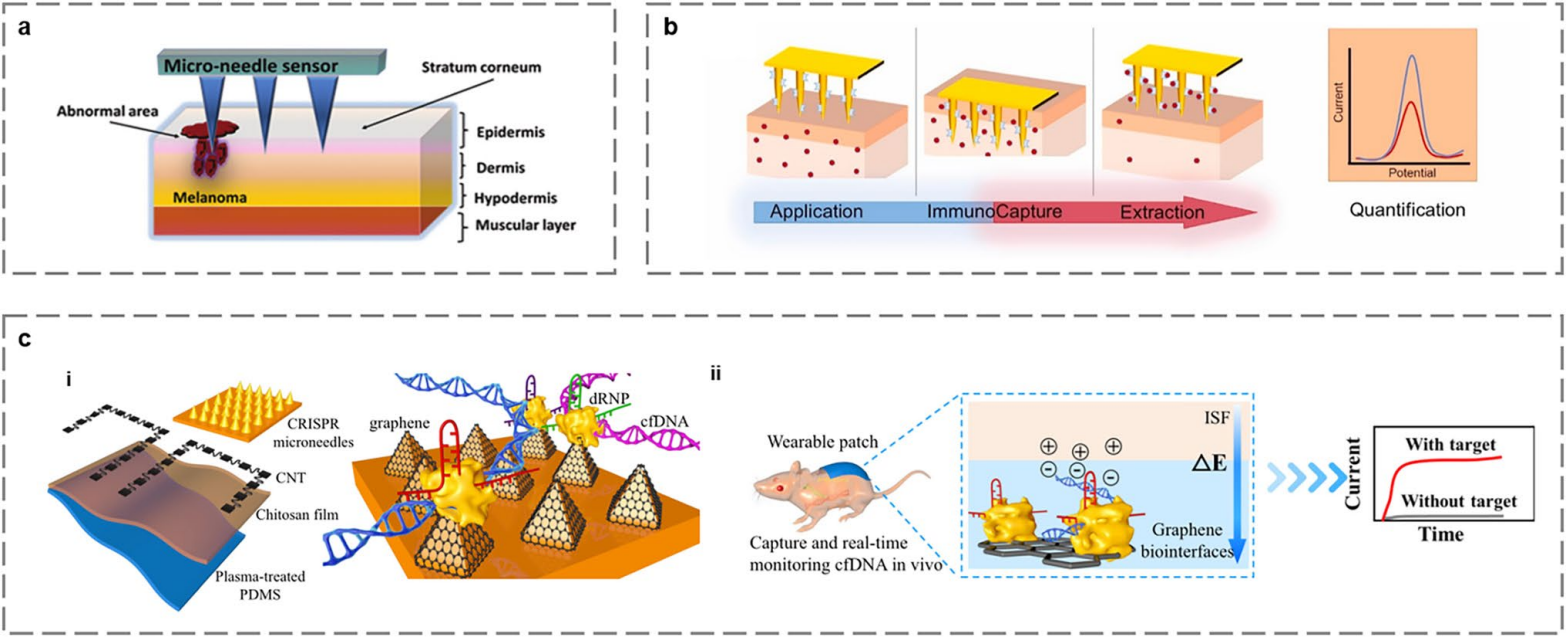
Schematic of MN-based electrochemical microsensors for monitoring biomacromolecules. **a** Schematic of monitoring of TYR melanoma biomarker using the MN sensor [97]. Copyright 2018 John Wiley and Sons. **b** Detection process of MNA-based electrochemical microsensors [98]. Copyright 2021 Elsevier. **c** Schematic of CRISPR MNA for extraction and real-time in vivo monitoring of cfDNA. (i) Scheme showing the fabrication of CRISPR MNA. (ii) Real-time monitoring of the enriched cfDNA in vivo [28]. Copyright 2022 Springer Nature

Proteins are fundamental organic substances in cells and play a crucial role in life's activities. Tyrosinase (TYR) is a polyphenol oxidase involved in melanin synthesis. Overexpression and accumulation of TYR in skin cells can lead to cutaneous melanoma [96]. Ciui et al. developed a minimally invasive MN electrochemical sensor that can be used for rapid screening of melanoma in the skin and deep tissue (Fig. 6a). MN devices were filled with catechol-coated carbon paste (CP), where immobilized catechol was rapidly converted to benzoquinone in the presence of TYR biomarkers and monitored by the sensor [97]. Dervisevic et al. prepared a high-density gold plated silicon microneedle array (Au–Si-MNA) as a sensing platform, and achieved the extraction and electrochemical monitoring of epidermal growth factor receptor 2 (ErbB2), a biomarker of breast cancer [98], shown in Fig. 6b.

#### Nucleic Acids

Nucleic acids are large biomolecules typically found within cells. There are two major types of nucleic acids: deoxyribonucleic acid (DNA) and ribonucleic acid (RNA) [99]. Nucleic acids are present in all plant and animal cells, microorganisms, viruses, and phages. They are vital substances in life, playing a significant role in determining growth, inheritance, mutation, and other biological phenomena. Clustered regularly interspaced short palindromic repeats (CRISPR) technology has been used in the rapid analysis of nucleic acids due to its precise gene editing capabilities [100]. Building on previous studies that combined MNA and counterion electroosmosis [12], Yang et al. reported a graphene platform on CRISPR-Cas9-activated conductive MNA [28]. The anode and cathode of the CNT pattern served as a reverse ion dialysis chamber, which was inkjet deposited onto a PDMS membrane modified with chitosan to separate negatively charged compounds such as nucleic acids or ascorbic acid and enrich cell-free DNA (cfDNA). The graphene biological interface and Cas9/sgRNA (dRNP) were integrated into the conductive MN of the working electrode to provide effective charged compound interactions and electron transport. Finally, the conductive CRISPR MNA as the working electrode was connected to the anode side of the CNT pattern, enabling it to continuously search and recognize the target cfDNA (Fig. 6c–i). The platform enabled real-time monitoring of viral, septic, and cfDNA with 60% fetal bovine serum resistance to interference, and in vivo studies in mice demonstrated the stability of the microsensor for more than 10 days. The device has a lot of potential for long-term in vivo monitoring of cfDNA and might be utilized for illness screening and prognosis. (Fig. 6c–ii).

Monitoring of biomacromolecules in animals helps to identify disease risk factors early and take corresponding prevention and intervention measures. By regularly monitoring the levels of specific proteins or nucleic acids, early signs of potential diseases can be identified, and preventive measures can be taken in advance to reduce the risk of disease occurrence.

### Electrolytes

Cations of alkali metals (Na^+^, K^+^) and basic (Mg^2+^, Ca^2+^) earth metals, as well as anions such as Chloride ion (Cl^−^), are essential for cellular physiology. Maintaining strict control over intracellular and extracellular ion concentrations is essential for regulating cell volume through osmotic pressure [101].

**Fig. 7:**
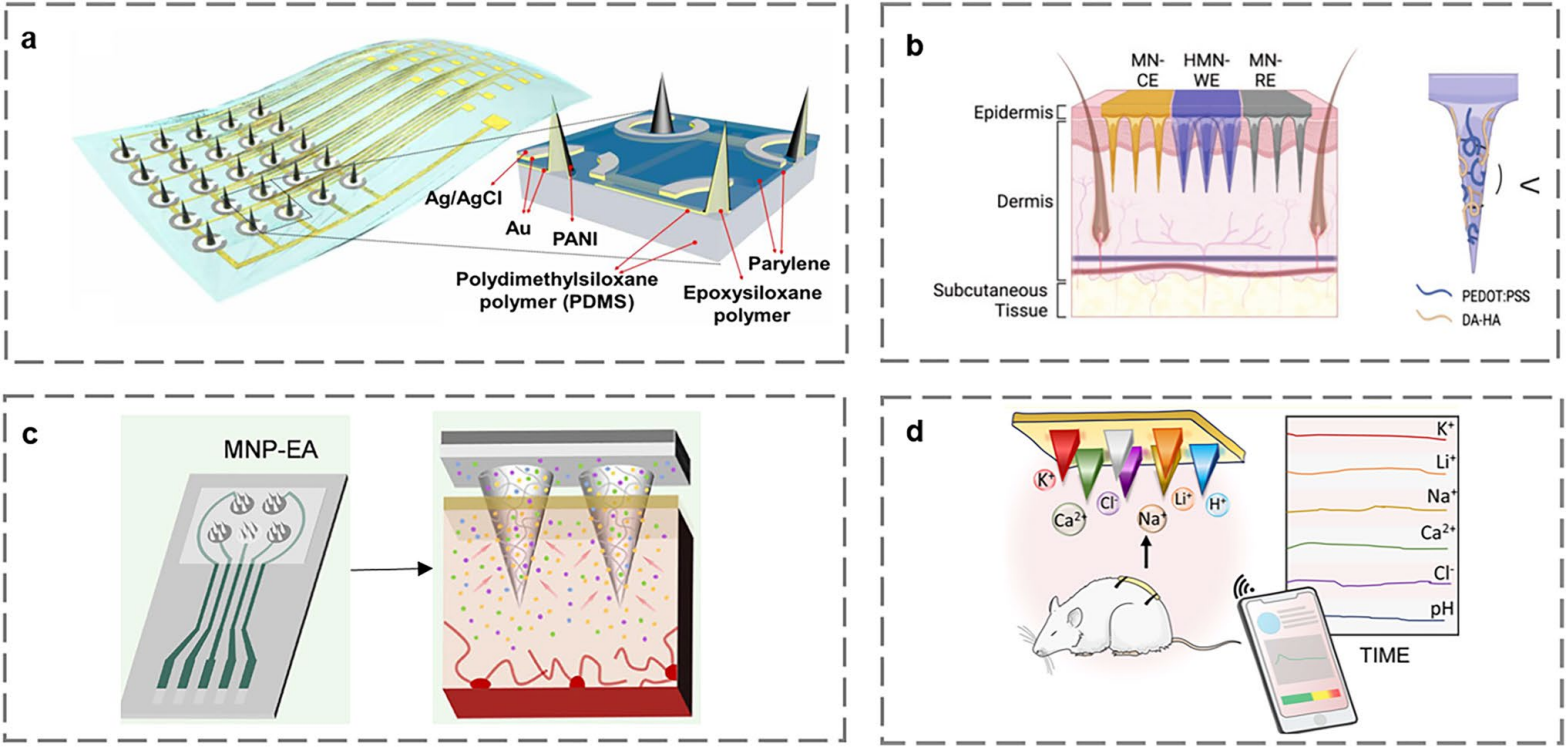
Schematic diagram of MN-based electrochemical microsensors for the monitoring of electrolytes. **a** Structural schematic of a flexible pH sensor array with MNA [13]. Copyright 2021 The American Association for the Advancement of Science. **b** Schematic of the structure of HMN-based electrode [103]. Copyright 2022 John Wiley and Sons. **c** MN-based potentiometric sensing system for simultaneous Na^+^, K^+^, Ca^2+^, and pH monitoring in vivo [107]. Copyright 2023 American Chemical Society. **d** A potentiometric MN-based sensor for in vivo multi-analyte monitoring [60]. Copyright 2022 American Chemical Society

Among various biomarkers, monitoring H^+^ concentration or pH levels is critical, as pH is vital for acid–base regulation and maintaining body homeostasis [102]. Physiological pH monitoring is clinically important as a predictor of diseases such as ischemia, multiple sclerosis, and peripheral arterial disease. Lee et al. reported an array of MN pH sensors based on PDMS (Fig. 7a). The MNA consists of silicone-based siloxane polymers with a high Young's modulus for better skin puncture and a low Young's modulus for conformability to the skin [13]. MNA with Au electrodes and polyaniline (PANI) deposited on the surface was used to monitor the pH distribution in the skin layer of a rat model of peripheral vascular disease. Considering better biocompatibility, Odinotski et al. prepared an HMN-based electrode (Fig. 7b) consisting of DA-HA hydrogel combined with PEDOT: PSS, with the intrinsic catechol quinone chemistry of DA used to measure the pH of ISF. HMN pH sensor is capable of in vivo measurements with 93% accuracy, providing a new direction for wearable sensors [103]. Li et al. designed a simple electrochemical microbial sensor (ECMB) based on the in situ self-assembly of AgNPs coated on MXene Titanium Carbide (Ti_3_C_2_T_x_) [104]. The proposed ECMB shows promise for high selectivity, accuracy, and repeatability in real-time monitoring of Cl^−^ in the brains of mice.

Fluid and electrolyte imbalance is a significant clinical issue that is directly associated with morbidity and mortality [105]. Electrolyte imbalance often involves multiple ions. Zhao et al. proposed a bifunctional electrochemical biosensor for simultaneous monitoring of glutamate and Ca^2+^ [106]. The bifunctional ME consisted of a Pt ME co-modified by glutamate oxidase (GluOx) and PtNPs for glutamate monitoring, along with a fully solid-state Ca^2+^-selective microelectrode (Ca-ISME) as a potential recording channel for Ca^2+^. The sensor exhibited excellent selectivity and limit of detection (LOD), and successfully recorded the dynamic changes in glutamate and Ca^2+^ in ischemic and normal mice. Zhu et al. created an MN sensor that is integrated with screen-printed electrodes and can monitor pH, Na^+^, K^+^, and Ca^2+^ (Fig. 7c) [107]. When MN penetrates the skin, ISF is quickly attracted to the electrode modified with the ion-selective membrane (ISM), causing the open circuit potential to change in a concentration-dependent manner. The sensor successfully monitored dietary-induced electrolyte imbalance in mice, with the potential for application in pet electrolyte monitoring. Molinero-Fernande et al. proposed an MN electrochemical sensing system for multi-analyte monitoring of pH, Na^+^, K^+^, Ca^2+^, Li^+^, and Cl^−^ (Fig. 7d) [60]. Selective detection of ions is achieved by modifying f-MWCNT and ion-selective membrane by dropwise addition on the MN surface of stainless steel substrate. In vivo experiments demonstrated good sensitivity, selectivity, and stability, although some differences in ion concentrations between ISF and blood require further investigation.

Implantable electrochemical sensors have been extensively used in animal health monitoring research. These sensors can detect various biological indicators, such as glucose, lactate, DNA, and proteins, and can monitor the changes in these indicators in real-time in the body, which is of great significance for the dynamic monitoring of animal health. In recent years, the development directions of implantable electrochemical microsensors for monitoring chemical substances in animal body fluids include multi-analyte monitoring, wireless remote monitoring, long-term stability, miniaturization, and biocompatibility. At the same time, there are issues with sensors such as stability, biocompatibility, data processing, energy supply, and cost to be addressed.

### Neurotransmitters

Neurotransmitters are essential chemicals that amplify, transmit, and transform signals in the brain [109]. Changes in neurotransmitter concentrations may lead to several brain diseases such as Alzheimer's disease (AD) [110] and Parkinson's disease (PD) [111]. Therefore, monitoring neurotransmitters has significant implications for understanding pathological mechanisms and improving prevention strategies. The basic structure and function of the animal nervous system and the human nervous system are similar, and many neural substances and pathways are highly conserved between animals and humans. Therefore, by studying animals’ neurotransmitters, we can gain a better understanding of the function and disease mechanisms of the human nervous system.

#### Dopamine

**Fig. 8:**
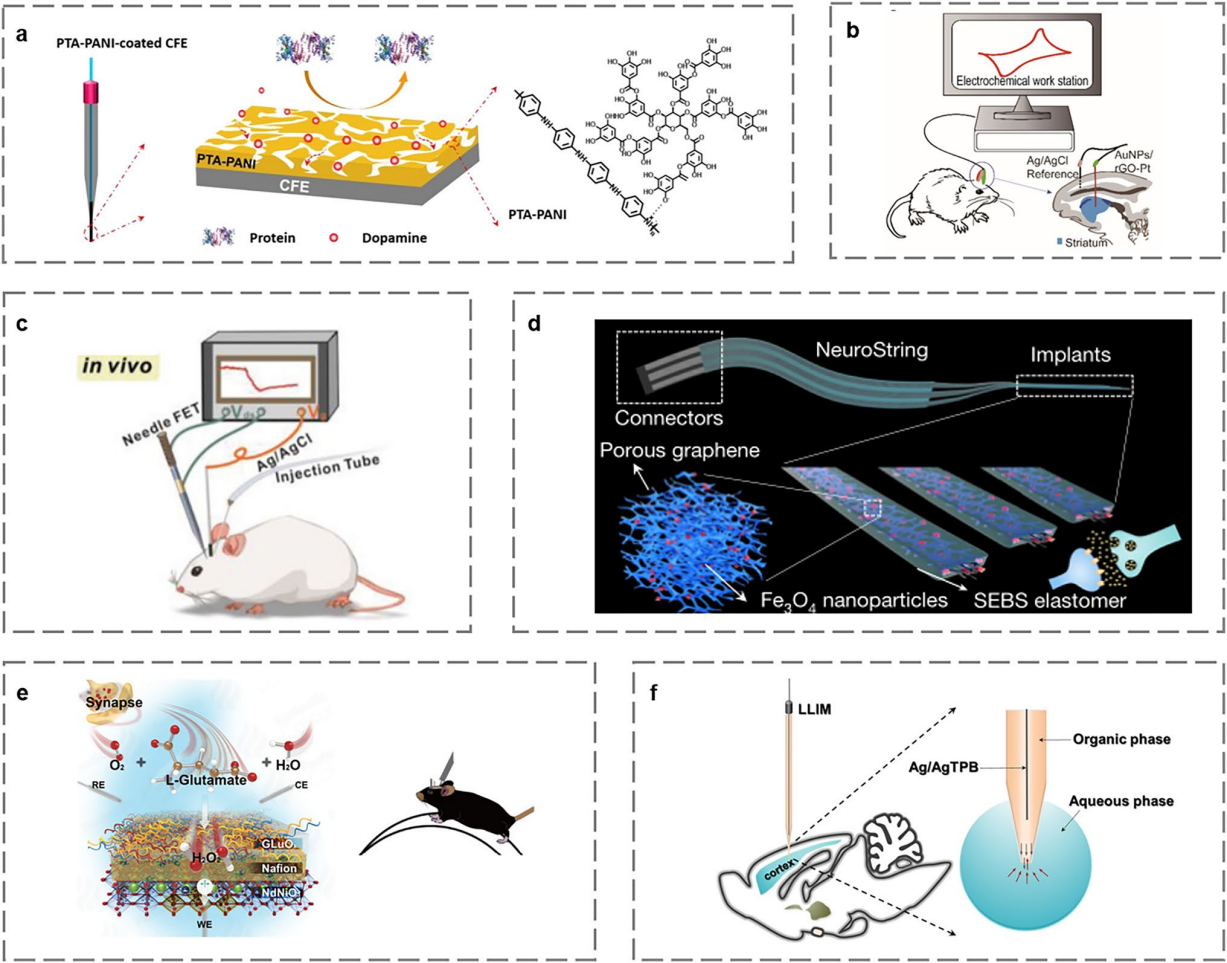
Schematic of implantable electrochemical microsensors for monitoring neurotransmitters. **a** An illustration of the structure of PTA-PANI-coated CFE [119]. Copyright 2019 American Chemical Society. **b** Schematic of AuNPs/rGO-Pt for in vivo electrochemical monitoring of DA [122]. Copyright 2019 Elsevier. **c** Schematic of the application of needle FET [20]. Copyright 2022 John Wiley and Sons. **d** Schematic illustration of the soft implant for sensing neurotransmitters in the brain [15]. Copyright 2022 Springer Nature. **e** Schematic of glutamate sensing with the nickelate-Nafion sensor [125]. Copyright 2020 American Chemical Society. **f** Illustration of the measurement with the LLIM in the rat brain [127]. Copyright 2021 American Chemical Society

Neurotransmitters can be classified chemically into biogenic amines, amino acids, and choline [112]. In vivo electrochemical studies using MEs have long been used to investigate neurotransmitters, providing real-time insights into their function in the brain and body [113]. Dopamine (DA), an important neurotransmitter in the central nervous system, is mainly found in the hypothalamus and pituitary gland. Abnormal DA concentrations may contribute to diseases such as Alzheimer [114], PD [115], and schizophrenia [116]. In vivo electrochemistry of carbon-fiber electrode (CFE) is one of the most useful methods for tracking neurochemicals in specific brain regions. Tang et al. first used CFE to quantify single DA. Intrasynaptic DA release and were surprised to find that harpagide (a natural product) could both enhance synaptic DA release and restore normal levels of DA release from damaged neurons, adding hope for Parkinson's treatment and prevention [117]. Since DA itself or the reactant can easily form a film on the CFE surface, unmodified CFE will inevitably suffer from surface biofouling and lead to a significant decrease in sensitivity [118]. To address this issue, Feng et al. prepared CFE modified by a nano-conducting PANI mixed with poly tannic acid (pTA) (Fig. 8a) and experimentally showed that the electrode has good antifouling properties, higher sensitivity than the unmodified electrode as well as excellent enrichment ability for DA electrochemical measurements [119]. Carbon-based nanomaterials are often used to modify the electrode surface of DA electrochemical sensors, which typically have high electrical conductivity, are biocompatible, and have a large specific surface area [120]. Taylor et al. studied CFE coated with a highly conductive PEDOT/CNT coating that exhibited extremely high sensitivity and selectivity for DA [121]. Metal ME is also used for in vivo monitoring of DA due to its high electrochemical catalytic activity and excellent conductivity. Chen et al. used gold nanoparticles (AuNPs) and reduced graphene oxide (rGO) modified Pt wires for in vivo electrochemical monitoring of DA (Fig. 8b). The modified electrodes exhibited high sensitivity, selectivity, and resistance to interference for DA, which was validated by implantation into the rat striatum. This experimental electrode has promising future applications for monitoring various neurotransmitters in vivo [122]. In addition, acupuncture needles' ability to be used as sensing devices for in vivo electrochemical monitoring of various biomolecules has been well shown. Zhou et al. fabricated a unique needle field-effect transistor (FET) microsensor based on an acupuncture needle (Fig. 8c), and it is shown to be capable of monitoring DA as well as neuropeptide Y in vivo [20]. The above work is excellent which gives a new insight into the FET domain and broadens the range of real-time in vivo detection species.

#### Serotonin

Many psychological disorders, such as depression, and anxiety, are related to low serotonin levels. Therefore, model experiments conducted on animals will help to explore the mechanism of psychological disorders. Fast scanning cyclic voltammetry (FSCV) is an electrochemical technique with precise temporal resolution and sensitivity for the measurement of fast neurotransmitter kinetics in vivo [123]. In a study by Abdalla et al., a novel method for stimulating and measuring serotonin concentrations was characterized in three brain regions [124]. It was shown that stimulation of axons leads to the production of serotonin in the three measured regions of the brain. Recently, Bao's team developed a stretchable, tissue-mimicking neurochemical bio-interface, NeuroString (Fig. 8d) [15]. The NeuroString sensor allowed real-time, multichannel, and multiplexed monoamine sensing in the brains of normally behaving mice, as well as the measurement of serotonin dynamics in the gut in the absence of undesired stimuli and interference with peristaltic motility. The microsensor has the potential to be used as a biomolecular sensing for soft organs throughout the body.

#### Glutamate

Glutamate is one of the most abundant and predominant excitatory neurotransmitters in the central nervous system, accurate monitoring of excitatory neurotransmitter levels is critical for understanding the underlying neuronal processes of various illnesses. Sun et al. created and evaluated a new calcium titanate-nickelate-Nafion electrode for recording glutamate release from electrically activated brain slices in vitro and primary visual cortex (V1) in vivo in awake mice exposed to visual stimuli (Fig. 8e) [125]. The electrode was shown to have high selectivity for glutamate, a fast response time (1.2 s) and LOD (16 nM). Recently, Chu et al. developed a flexible electrochemical microsensor for simultaneous monitoring of L-glutamate and GABA levels, which are anticipated to play a vital role in the excitation: inhibition balance [126]. The flexible electrodes were electrochemically deposited with Pt to increase the electrochemically active surface area, and in vitro and in vivo experiments in mice demonstrated their accuracy for long-term monitoring.

#### Choline

Choline is another neurotransmitter that participates in the transmission of neural signals and plays an important role in motor control, memory, and other aspects. Wang et al. developed a liquid/liquid interface microsensor (LLIM) (Fig. 8f) for monitoring the redox inactive neurochemical choline (Ch) in the rat brain [127]. Ch was found at a specific ion transfer potential and a unique ion transfer current signal based on the difference in the solvation energy of choline in cerebrospinal fluid (aqueous phase) and 1,2-dichloroethane (organic phase). LLIM responded well to choline, with strong linearity and selectivity with a LOD of 0.37 µM. For the first time, it was demonstrated that LLIM can be employed in the brain.

Overall, these advancements in neurochemical sensing techniques provide valuable insights into the functioning of neurotransmitters and their role in various diseases and physiological processes. The development of these techniques not only helps us better understand the working principles of the nervous system, but also provides researchers with more tools to explore the relationship between neurotransmitters and diseases.

### Oxidative Stress Metabolites

Small molecule products of oxidative metabolism have a substantial impact on physiological processes and pathological pathways. Reactive oxygen species (ROS) and reactive nitrogen species (RNS) are two examples of significant metabolites [113]. They play diverse roles in the regulation of several physiological processes at relatively low concentrations.

#### H_2_O_2_

**Fig. 9:**
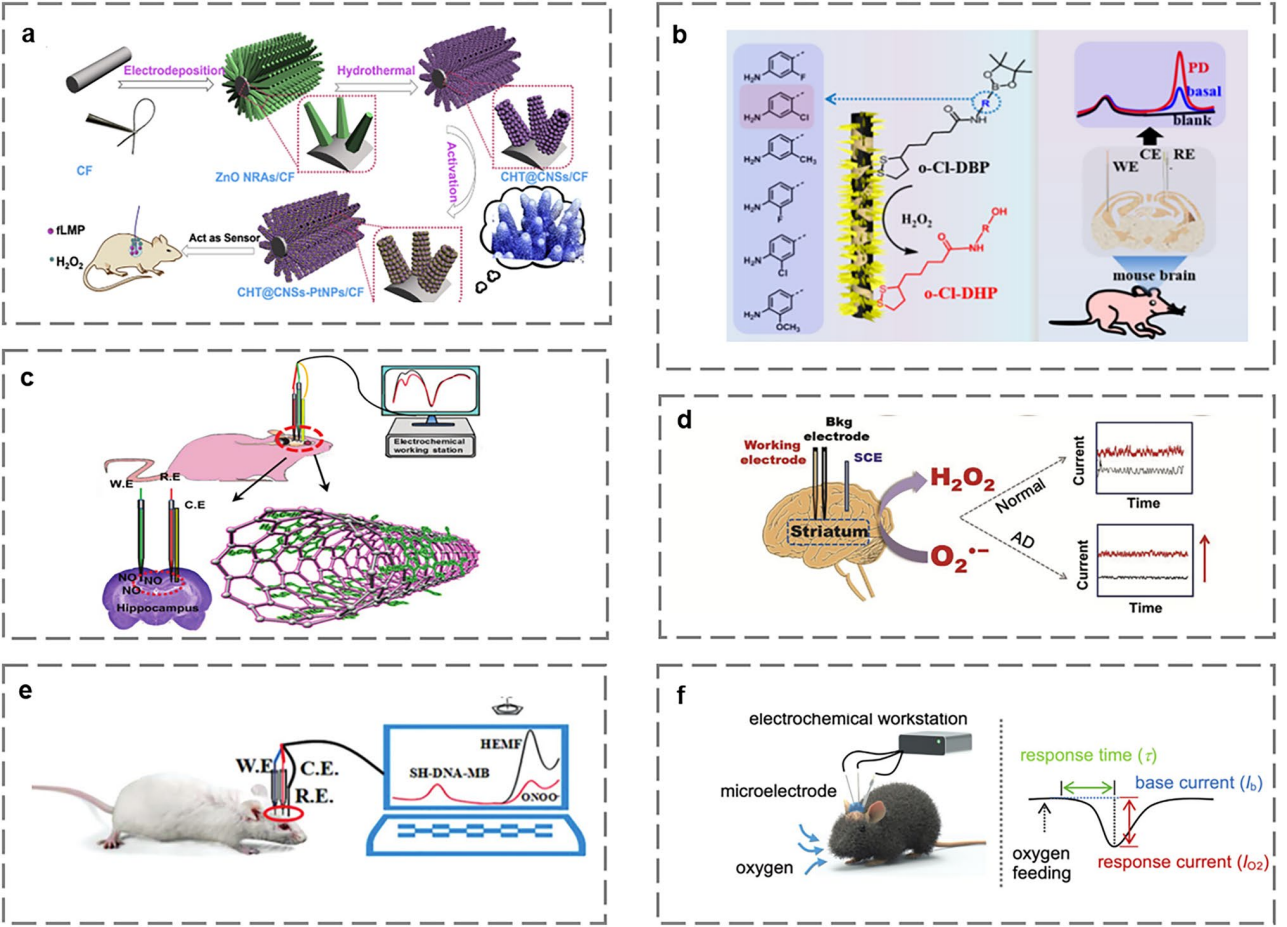
Schematic diagram of implantable electrochemical microsensors for monitoring oxidative stress metabolites. **a** Schematic illustration of the synthesis of coral-like hierarchical structured HCT@CNSs-PtNPs/CF ME [108]. **b** An electrochemical microsensor for in vivo monitoring of H_2_O_2_ in PD mouse brain [128]. Copyright 2022 American Chemical Society. **c** A schematic of ratiometric electrochemical sensor for real-time monitoring of NO in a rat brain following global cerebral ischemia [129]. Copyright 2017 Royal Society of Chemistry. **d** Schematic diagram showing the fabrication of SOD/PIL/PB/CNT modified sensor for the determination of O_2_^•−^ [132]. Copyright 2019 Elsevier. **e** Schematic of electrochemical biosensor for monitoring of ONOO^−^ in the rat brain [134]. Copyright 2019 Royal Society of Chemistry. **f** Diagram of a three-electrode device used for in vivo electroanalysis in the brain of an anesthetized mouse [137]. Copyright 2022 John Wiley and Sons

Overproduction of ROS/RNS contributes to the development of cardiovascular disease, cancer [63, 108]. Zhang et al. synthesized and designed a nano-hybrid ME based on a coral-like layered structured carbon nano-scaffold modified by high-density and ultrafine PtNPs (Fig. 9a). The sensor was capable of monitoring H_2_O_2_ generated from different types of live cells and in cancer tissue from laboratory mice [108]. Oxidative stress can also affect the synthesis and release of neurochemicals, further affecting the function of the nervous system. Therefore, the simultaneous monitoring of oxidative stress products and neurochemicals in the study of nervous system disease can provide a more comprehensive understanding of the pathophysiological process of the nervous system. This subsection provides a brief summary of monitoring for several typical ROS/RNS. Recently, Luo et al. metered a series of 5-(1,2-dithiolan-3-yl)-N-(4-(4,4,5,5-tetramethyl-1,3,2-dioxaborolan-2-yl)-phenyl)-pentanamide (DBP) derivatives, which were used as probes for specific reactions with H_2_O_2_ (Fig. 9b) [128]. The designed electrochemical microsensor provides a ratiometric strategy to track H_2_O_2_ in real time with high selectivity and accuracy and was successfully applied to the measurement of H_2_O_2_ in the brains of mice with PD.

#### Nitric Oxide

Nitric oxide (NO) is an important free radical synthesized and released by brain cells. At low (millimolar) levels, it regulates synaptic transmission and neuronal activity, but at much higher levels, mediates neuronal damage through oxidative stress. Liu et al. developed a simple ratio electrochemical biosensor using heme-modified carbon nanotube fibers (CNFs) (Fig. 9c), which use Nafion to generate a biofilm layer to improve sensitivity and selectivity for monitoring NO in the brain of rats after cerebral ischemia [129]. However, Nafion cannot completely prevent the unnecessary oxidation of endogenous redox molecules. Meiller et al. designed a micro sensor coated with an innovative shielding layer of fluorinated dry gel and applied it to NO monitoring [130]. Compared with the sensor coated with Nafion, the fluorinated dry gel layer can protect the electrode from the damage of oxide derivatives, and has excellent selectivity and specificity. The fluorinated dry gel also shows potential antifouling ability, which is critical for in vivo quantification.

#### Superoxide Anion

Superoxide anion (O_2_^•−^) is an important ROS in the brain system and is associated with the development of many neurological diseases, including AD [131]. Peng et al. coated functionalized ionic liquid polymer (PIL) onto PB nanoparticles (PBNPs) and carbon CNTs [132]. PIL served as a substrate for the immobilization of superoxide dismutase (SOD). Modified CFE successfully monitored O_2_^•−^ concentration changes in the living brains of normal and AD model rats (Fig. 9d). Peroxynitrite (ONOO^−^) is a reactive oxidant produced by nitric oxide and superoxide that reacts with proteins, lipids, and DNA to promote cytotoxic and pro-inflammatory responses [133]. Liu et al. developed an organic molecule, 4-(S-(6-mercaptohexyl)benzothioate-6-yl)-7-(diethylamino)-2-(4-(piperazinyl diferroformamide-1-yl)phenyl)chromenylium (HEMF), with pyridine group and ONOO^−^ specific reactions in HEMF, for the assessment of ONOO− levels in hippocampus, striatum, and cortex of rat brain after cerebral ischemia, enabling real-time quantitative analysis (Fig. 9e) [134].

#### O_2_

O_2_ is a critical indicator of neuronal activity in neurochemical processes, and abnormal fluctuations of O_2_ in the brain can interfere with neurochemical processes and even lead to neurological dysfunction [135]. Cao et al. prepared an electrochemical microsensor for real-time monitoring of O_2_ in the brain by electrophoretically depositing Co/N/C catalysts on CFE [136]. Co/N/C nanocomposites catalyze oxygen reduction reactions without producing toxic intermediates and have good selectivity and sensitivity to O_2_. Recently, Su's team combined in vivo electrochemistry with physiological and behavioral analysis to analyze oxygen metabolic depletion in the brain [137], designed biocontamination-resistant CFE to measure in vivo currents to obtain oxygen levels (Fig. 9f). Simultaneous recording of mouse physiology and behavior to explore the correlation between oxygen levels and mouse behavior showed that metabolic oxygen consumption in brain regions is not independent of each other, but involves collaborative control and interactions.

The excessive production of oxidative stress products can lead to some physiological diseases, including but not limited to cardiovascular diseases, neurodegenerative diseases, cancer, etc. The monitoring of oxidative stress products has profound significance for the study of physiological disease mechanisms.

### Others

Electrochemical microsensors can also be used for monitoring of therapeutic drugs in model experimental animals to facilitate the treatment of human diseases. Goud et al. reported an MN sensing platform for continuous minimally invasive orthogonal electrochemical monitoring of levodopa (L-Dopa), which is the most effective treatment for PD [138]. The real-time monitoring system provides built-in redundancy and different dimensions of information to selectively and sensitively track (L-Dopa) levels in vivo. Joshi et al. proposed an attractive MN sensing platform for monitoring opioid fentanyl in real serum samples [139]. The MN-based sensor showed the direct oxidation of fentanyl in liquid samples by using a highly sensitive square wave voltammetry (SWV) method. Antibiotics are widely used in medicine and the environment because they inhibit the growth of bacteria. However, antibiotic residues in the environment and food sources pose a great potential threat to animal health [140]. In future, it is expected to develop electrochemical sensors for monitoring antibiotic residues in animals.

Implantable microsensors have the following characteristics: They require long-term stability and biocompatibility; Need to be in contact with tissue and conduct high-precision measurements; It requires features such as small size, high throughput, high selectivity, and high stability. Due to the complexity and uncertainty of the body, there are still some challenges, such as the stability and interaction between electrodes and tissues, biological and immune reactions, flexibility and mechanical stability of electrodes and cables. The future development trends include better materials and manufacturing technologies, more complex signal processing and data analysis technologies, better energy management and wireless communication technologies, more refined neural control, and stimulation technologies, and so on. These development trends will make implantable sensors more effective, safe, reliable, and adaptable to a wide range of application scenarios, such as providing reliable animal disease models for the eventual conquest of human diseases.

## Conclusion and Future Perspectives

This review provides an overview of the fabrication and implantation technologies of implantable electrochemical microsensors and summarizes their application for monitoring physiological information in animals in vivo. Implantable electrochemical microsensors are minimally invasive, painless, and offer opportunities for continuous monitoring in vivo as well as long-term stability. However, in order to facilitate the further development and commercialization of implantable electrochemical microsensors, several important issues must be addressed.

1. *Protective mechanism and anti-interference coating of implantable electrochemical sensors* The host's foreign body reaction (FBR) can lead to the adsorption of certain proteins on the sensor's surface. This surface biofouling can cause equipment contamination, loss of specificity, and overall surface passivation [142]. Therefore, it is necessary to employ protective mechanisms to minimize these effects. Polyethylene glycol (PEG) and its derivatives [143], zwitterionic polymers [142, 144], amphiphilic phospholipid polymers [145], and other materials are widely used to protect sensors from pollution. It is necessary to employ protective mechanisms to minimize these effects. In addition, implantable sensors must exhibit good anti-interference performance to ensure accuracy and reliability.
2. *Long-term stability and specificity of sensitive materials* The sensitive materials used in sensors are typically biological molecules, such as enzymes. Long-term use may lead to the degradation or deactivation of these molecules, thereby reducing the sensitivity and specificity of the sensor. Although some researchers have attempted to use degradable materials to encapsulate electrodes and gradually expose enzymes, the outcomes have been limited [146]. Therefore, it is vital to investigate methods for enhancing the long-term stability of these biomolecules while preserving their selectivity.
3. *Sensitivity and instantaneous response resolution of sensors* Sensor sensitivity and response speed are crucial indications of sensor performance. To enhance sensor sensitivity, it is necessary to explore ways to improve signal acquisition and amplification. Similarly, researchers must investigate methods for optimizing the structure and response mechanism of sensors to improve their response time.
4. *Sensor array and thermal damage issues* Sensor arrays tend to be used to measure chemicals in the nervous system. However, implantable sensors may cause damage to surrounding tissue. Miniaturization of the electrode can mitigate the damage. At the same time, the implantation technology and fixation method of the implantable sensor can influence both the detection reliability and the extent of damage caused.
5. In vivo *energy supply issues* Due to recent technological developments, advances in wireless communication have enabled implantable microsensors to be untethered while in the body. However, a major challenge arises when these microsensor devices operate in vivo: limited energy supply [147]. Conventional energy supply is achieved through batteries. Researchers have discovered that different positions and organ systems in the body can harness various types of energy, such as mechanical energy from muscle contraction and chemical energy from biological molecules. Among them, mechanical energy can be divided into piezoelectric energy, frictional energy, and electromagnetic induction energy. Generators based on mechanical energy can convert the mechanical energy of animal movement into useful electrical energy, providing power for microsensors [148]. The use of chemical energy for power generation can be divided into primary cells and biofuel cells. The oxidation/reduction reactions of biological molecules can convert chemical energy into electrical energy to provide power for microsensors. Among the above applications, enzymatic biofuel cells show promise in generating electricity from biological catalytic reactions [149]. Enzymatic biofuel cells are capable of short-term and long-term implantation in mammals such as rats and rabbits [9, 150]. As a consequence, it is feasible to collect the available energy in the body and convert it into electrical energy to supply the microsensors in the body for long-term monitoring. Currently, there is not much research on this area, leaving room for further development.

In the foreseeable future, the advancement of implantable microsensor technology will offer enhanced capabilities for monitoring and comprehending the physiological condition of animals. Through regular monitoring of animal physiological data, we can improve disease diagnosis, assess the effects of environmental pollution and climate change on animal well-being, and develop and deploy less invasive and more efficient devices to gain deeper insights into the physiological state of animals. Research conducted on model experimental animals can provide valuable insights into complex disease mechanisms, including obesity, diabetes, and zoonotic diseases. Additionally, the integration of implantable electrochemical microsensors with artificial intelligence and Internet of Things (IoT) platforms offers the potential for real-time monitoring of animal diseases, visualization of health data, and timely implementation of disease prevention strategies.
